# The Mental State in Myxœdema

**Published:** 1904-06-25

**Authors:** 


### THE MENTAL STATE IN MYXŒDEMA.

H. Wolseley-Lewis,[1] from his experience with a number of myxœdematous patients at Banstead Asylum, contributes an analysis of the mental state of such patients. They are usually sent to asylums as cases of dementia or melancholia, but they display few of the characteristics of either condition. The essential change appears to be a deficient power of energising the motor-cells. Hence the patients are languid and tired after even slight exertion, and sit for hours in the same attitude. Their perceptive faculties are fairly acute, but the power to express their ideas is seriously minimised. Abnormal perceptions are not prominent, and are rather of the nature of "pseudo-hallucinations." Consciousness, as a rule, is clear, and the patients are aware of and troubled by their mental disability. Memory as regards retentiveness is good, but the capacity to receive impressions is deficient. There s a retardation of thought, and the elaboration of an idea is complete though slow. Judgment and reasoning are not disturbed when the attention is once aroused. But the ability to sustain attention and mental action is defective. Signs of disturbed emotion are rare with myxœdematous patients, but the affective state is not one of indifference, and affection for children or other relatives is retained. The essential departure from the normal is weakness of volition and lack of initiative. Hence even the simplest action requires a very special exercise of the will, and an external stimulus to produce an active response must be unusually strong. The difficulty is not in perception or understanding, but in action, and more especially in the starting of an action. In one patient it was observed that the knee-jerk, though well marked, was delayed, and the contraction of the muscle was unduly prolonged. This restriction of the deficient mental state to the sphere of action suggests either that some toxin inhibits the nerve processes which originate a motor impulse, or that the absence of some substance from the blood interferes with the discharge. Whether one supposition or the other is correct the condition is met by the administration of thyroid, and the good effect of this appears to be increased by the simultaneous prescription of syrup of iodide of iron. The majority of the cases are sent to asylums in error, and could be equally well treated at home.

[1] Lancet, April 23, 1904.

